# Oesophageal Adenocarcinomas: Where Do We Stand Today?

**DOI:** 10.3390/cancers13010109

**Published:** 2020-12-31

**Authors:** Karl-Frederick Karstens, Björn Ole Stüben, Matthias Reeh

**Affiliations:** Department of General, Visceral and Thoracic Surgery, University Medical Center Hamburg-Eppendorf, 20246 Hamburg, Germany; k.karstens@uke.de (K.-F.K.); b.stueben@uke.de (B.O.S.)

Oesophageal cancers (oesophageal squamous cell carcinomas and adenocarcinomas) haven been responsible for more than one million deaths worldwide in 2018 [1]. Of note, the incidence of oesophageal adenocarcinomas (OACs) has increased greater than six-fold over the last decades and five-year survival rates are still around 20% despite advances in the multimodal treatment [2,3].

The only known precursor for OACs is Barrett’s oesophagus [4]. Barrett’s oesophagus (BO) is defined as replacement of the squamous epithelium of the oesophagus by intestinal-type simple columnar epithelium. It has been proposed that these mucosal alterations further progress to dysplastic lesions and ultimately to OACs. In a meta-analysis of 29 studies of BO, the incidence rate of malignant transformation was 5.3 per 1000 person years, when early cancers were included [5]. However, the prevalence of BO is still unknown, but it is estimated to effect two to seven percent of the people [6]. Several risk factors like male sex, increasing age, gastro-oesophageal reflux disease and obesity have been shown to be positively associated with the progression of BO to OACs [7]. How these risk factors lead to OACs remains under discussion [8]. One explanation is the inflammatory microenvironment. Several studies have described a shift within the composition of the local immune cells in BO leading to the development of OACs [9,10]. However, why only certain people with BO develop OACs warrants further research, as the majority of people with BO show no progress to OACs.

There is a major lack of knowledge about the development of OACs. Furthermore, the sufficient classification of OACs remains a clinical challenge. Oesophageal adenocarcinomas predominantly occur in the lower third of the oesophagus at the oesophago–gastric junction (Z-line). The tumor localization is defined in relation to the Z-line by endoscopy, which greatly depends on the expertise of the endoscopist. In addition, an internationally standardized classification of these junctional tumors is still missing. The Siewert classification divides junctional carcinomas in three subtypes according to the epicenter of the tumor in relation to the Z-line, while the classification system of the American Joint Committee on Cancer (8th edition) uses only two subtypes [11,12]. Many consider adenocarcinomas distal of the Z-line to be of gastric origin (Siewert type III) while tumors above the Z-line as genuine oesophageal cancers (Siewert type I). However, tumors centered right at the Z-line (Siewert type II) cannot be easily allocated to any of the latter. This is of relevance, since the lymphatic spread towards the mediastinum seems to be different between tumors with gastric and oesophageal origin. This challenges the surgeon to select the proper surgical approach (transhiatal or thoraco-abdominal esophagectomy) to perform a sufficient lymphadenectomy [13]. Hence, further studies are needed to clarify the biological behavior of these specific tumors.

While the use of perioperative chemotherapy has been generally accepted in the treatment of locally advanced oesophageal adenocarcinomas [14,15], the possible benefit of neoadjuvant radiochemotherapy is currently investigated [16,17]. However, poor survival rates are still in need of improvement. Thus, new therapy approaches have emerged, that do not target the tumor cells directly but rather modulate the response of the immune system. These immune therapies target immune checkpoints like the PD-1/PD-L1 axis and the cytotoxic T-lymphocyte antigen 4 (CTLA4), which regulate T-cell activation and thereby direct the immune response against the carcinoma. Thus, monoclonal antibodies (e.g., nivolumab and pembrolizumab: anti-PD1 and ipilimumab: anti-CTLA4), that inhibit these checkpoints, stimulate antitumor immunity and might lead to an ongoing tumor regression [18,19]. Interestingly, the combination of anti-PD1 and anti-CLTA4 antibodies led to an increased immune response. However, nearly half of the patients treated with this combination experienced severe toxic side effect and treatment-related adverse events, resulting in a discontinuation of treatment in 20% of the patients [18]. Of note, an increased response rate was reported in patients with microsatellite unstable (MSI) tumors [18,20]. In addition, the PD-L1 combined positive score (CPS) has also been used to predict a response for anti-PD1 therapy [21].

In a recent study, including only gastric and gastro-oesophageal junctional adenocarcinomas with a CPS of ≥1 non-inferiority and with a CPS of ≥10, a clear survival benefit for pembrolizumab monotherapy compared to standard-of-care chemotherapy was observed. Interestingly, no additional survival effect was observed when chemotherapy and immune therapy were combined [22]. However, due to the complexity of the tumor-immune interactions, the described biomarkers (MSI and CPS) appear to be insufficient to safely select patients for these therapies, define the optimal time of treatment or warrant the use of a combinational therapy. Thus, more studies are needed to help detect further potential biomarkers or define more accurate cut-off levels, which may correlate to immunotherapy outcome. It must be taken into account that many factors can influence the effect of the immune therapy, like the microenvironment and environmental factors, the microbiome and the heterogeneity of the OACs, which might lead to a resistance of a selected therapy.

In conclusion, OACs are becoming a major public health issue. However, an internationally standardized classification system is still missing. This is needed to generate highly comparable data to further investigate OACs and expand the number of possible individualized treatment options, especially in response to rapidly emerging immune therapies.
